# Towards consistent generation of pancreatic lineage progenitors from human pluripotent stem cells

**DOI:** 10.1098/rstb.2014.0365

**Published:** 2015-10-19

**Authors:** Maria Rostovskaya, Nicholas Bredenkamp, Austin Smith

**Affiliations:** Wellcome Trust-Medical Research Council Stem Cell Institute, University of Cambridge, Tennis Court Road, Cambridge CB2 1QR, UK

**Keywords:** embryonic stem cells, differentiation, pancreatic progenitors

## Abstract

Human pluripotent stem cells can in principle be used as a source of any differentiated cell type for disease modelling, drug screening, toxicology testing or cell replacement therapy. Type I diabetes is considered a major target for stem cell applications due to the shortage of primary human beta cells. Several protocols have been reported for generating pancreatic progenitors by *in vitro* differentiation of human pluripotent stem cells. Here we first assessed one of these protocols on a panel of pluripotent stem cell lines for capacity to engender glucose sensitive insulin-producing cells after engraftment in immunocompromised mice. We observed variable outcomes with only one cell line showing a low level of glucose response. We, therefore, undertook a systematic comparison of different methods for inducing definitive endoderm and subsequently pancreatic differentiation. Of several protocols tested, we identified a combined approach that robustly generated pancreatic progenitors *in vitro* from both embryo-derived and induced pluripotent stem cells. These findings suggest that, although there are intrinsic differences in lineage specification propensity between pluripotent stem cell lines, optimal differentiation procedures may consistently direct a substantial fraction of cells into pancreatic specification.

## 1. Introduction

Human pluripotent stem cells (PSC) represent a renewable source of differentiated cell types for fundamental and applied research and potentially for use in cell-based therapies including for type I diabetes. Moreover, generation of human induced pluripotent stem cells (hiPSC) from somatic cells opens the possibility of producing patient-specific cells for autologous transplantation [1,2]. However, to realize their potential for study of beta cell biology and immunology, and for cell therapy applications, generically applicable methods are needed for efficient differentiation to pancreatic lineages.

Multiple protocols have been described for converting PSC to pancreatic progenitors *in vitro* [3–11]. *In vivo* development of the pancreas is preceded by specification of definitive endoderm (DE) [12–14]. The main inducer of DE in the vertebrate embryo and during *in vitro* differentiation from PSC is Nodal signalling [15,16], which can be simulated by high doses of other TGF*β* family members such as Activin A or GDF8 [17,18]. This process also requires transcriptional activation by beta-catenin [15,19], which can be stimulated by Wnt3a or by chemical inhibition of GSK3*β*. Additional signals such as bone morphogenetic proteins (BMPs), fibroblast growth factors (FGFs) and inhibition of PI3K/Akt may also contribute to DE specification [4,19,20], although their precise modes of action remain undefined.

The DE forms primitive gut, which undergoes patterning along the anterior–posterior axis to establish foregut, midgut and hindgut domains instructed by local signalling cues, including Wnt, BMP, FGF and retinoic acid [21]. Further, guided by a combination of signals received from neighbouring tissues [22], cells located at the junction of foregut and midgut become specified to the pancreatic anlage and evaginate to form the dorsal and ventral pancreatic buds [23]. At this stage, pancreatic epithelial cells represent early multipotent progenitors, which later form all lineages in pancreas—acinar, ductal and endocrine [24,25]. They express and are critically dependent on the transcription factor PDX1, pancreatic and duodenal homeobox 1. As its name implies, PDX1 is not exclusive to pancreas but is expressed in a region of posterior foregut including stomach and duodenum [26,27].

Based, in large part, on the elucidation of major signals operating during specification of pancreatic epithelium in the mouse embryo, methods have been devised to generate PDX1-expressing progenitors from PSC *in vitro* involving various combinations of factors and timings of treatment [3–5]. While the requirement for retinoic acid and BMP inhibition is well accepted, the role of FGFs [28] and Wnt [5] for *in vitro* induction has been challenged [4]. These apparent discrepancies are difficult to resolve because of the lack of reference data from the human embryo and because the existing protocols have not been evaluated side-by-side across a panel of PSC lines.

Here we systematically compared approaches for PSC specification to DE and further to PDX1-expressing presumptive pancreatic endoderm using both embryo-derived pluripotent stem cells (hESC) and hiPSC. Our data define conditions for reliable generation of pancreatic derivatives from different PSC.

## 2. Material and methods

For the detailed description of culture and differentiation protocols, see the electronic supplementary material, Supplementary Experimental Procedures.

### (a) Pluripotent stem cell lines and culture

hESC lines used in the study were H9 [29] and Shef6 [30]. Transgene-free hiPSC cells were derived previously in our laboratory from human fibroblasts (FiPS) and adipose tissue cells (AdiPS) by expression of OCT4, SOX2, KLF4 and cMYC using Sendai virus [31]. PSC were cultured either on feeder layers of *γ*-irradiated mouse embryonic fibroblasts in KnockOut Serum Replacement and FGF2 containing medium (KSR/MEF), or on Matrigel-coated plates (Corning) in Essential 8 medium (E8, Gibco Life Technologies).

### (b) Definitive endoderm differentiation

The DE differentiation was performed with H9 hESC and FiPS cells expanded in KSR/MEF or E8 conditions, according to the five methods summarized in table 1. Data were collected from six independent experiments.

**Table 1.** Conditions for definitive endoderm differentiation of human PSCs.

protocol	references	stage 1		
DE-1	Loh *et al*. [4]	100 ng ml^−1^ Activin A 100 nM PI103 3 µM Chiron 10 ng ml^−1^ FGF2 3 ng ml^−1^ BMP4 −1 day	100 ng ml^−1^ Activin A 100 nM PI103 20 ng ml^−1^ FGF2 250 nM DM3189 −2 days	
DE-2	Touboul *et al*. [20]	100 nM PI103 100 ng ml^−1^ Activin A 20 ng ml^−1^ FGF2 10 ng ml^−1^ BMP4 −3 days		
DE-3	Rezania *et al*. [7]	100 ng ml^−1^ GDF8 3 µM Chiron −1 day	100 ng ml^−1^ GDF8 0.3 µM Chiron −1 day	100 ng ml^−1^ GDF8 −1 day
DE-4	D'Amour *et al*. [15]	100 ng ml^−1^ Activin A 25 ng ml^−1^ Wnt3a −1 day	0.2% FBS 100 ng ml^−1^ Activin A −2 days	
DE-5	Cheng *et al*. [32]	100 ng ml^−1^ Activin A 40 ng ml^−1^ Wnt3a −1 day	0.5 ng ml^−1^ BMP4 10 ng ml^−1^ bFGF 100 ng ml^−1^ Activin A 10 ng ml^−1^ VEGF −4 days	

### (c) Pancreatic differentiation

For pancreatic differentiation from the DE stage, we employed six published protocols summarized in table 2. Data were collected from 12 independent experiments. The pancreatic progenitors were further differentiated to insulin-producing cells using a recently described protocol [7].

**Table 2.** Conditions for differentiation to PDX1-expressing progenitors.

		stage 2	stage 3	stage 4^a^	
protocol	references	primitive gut endoderm	PDX1+, presumptive pancreatic endoderm		validation of differentiation potential of the resulted cells
P-1	Kroon *et al*. [3]	2% FCS 50 ng ml^−1^ FGF7−3 days	2 µM ATRA 250 nM SANT-1 250 nM DM3189 −3 days	—	formation of polyhormonal cells *in vitro*; maturation *in vivo* to functional beta cells
P-2	Nostro *et al*. [5]	3 ng ml^−1^ Wnt3a 50 ng ml^−1^ FGF10 250 nm DM3189 −3 days	2 µM ATRA 250 nM SANT-1 250 nM DM3189 50 ng ml^−1^ FGF10 −3 days	—	formation of polyhormonal cells *in vitro*
P-3	Loh *et al*. [4]	250 nM DM3189 4 µM IWP2 500 nM PD0325901 2 µM ATRA −1 day	2 µM ATRA 250 nM SANT-1 250 nM DM3189 500 nM PD0325901 −3 days	—	not reported
P-4	Rezania *et al*. [7]/Pagliuca *et al*. [6]	250 µM ascorbic acid 50 ng ml^−1^ FGF7 −2 days	250 µM ascorbic acid 50 ng ml^−1^ FGF7 250 nM SANT-1 1 µM ATRA 100 nM DM3189 200 nM TPB −3 days	—	differentiation to monohormonal insulin+ cells *in vitro*; maturation *in vivo* to functional beta cells
P-5	Rezania *et al*. [7]			250 µM ascorbic acid 2 ng ml^−1^ FGF7 250 nM SANT-1 100 nM ATRA 200 nM DM3189 100 nM TPB −3 days	
P-6	Pagliuca *et al*. [6]			250 µM ascorbic acid 50 ng ml^−1^ FGF7 250 nM SANT-1 100 nM ATRA −5 days	

^a^Stage 4 conditions were applied only after protocol 4, constituting protocols 5 and 6.

### (d) Quantitative RT-PCR

Total RNA was extracted with RNeasy Mini Kit (Qiagen) and up to 1 µg was used for reverse transcription with SuperScript III (Invitrogen). Quantitative PCR was performed with TaqMan Fast Universal PCR Master Mix (Applied Biosystems) using Universal Probe Library (Roche) for detection. Primer sequences are listed in the electronic supplementary material, table S1. GraphPad Prism software was employed for data representation.

### (e) Flow cytometry

For surface marker staining, cells were dissociated using 0.5 mM EDTA and incubated with directly conjugated antibodies (electronic supplementary material, table S2) diluted in phosphate-buffered saline (PBS) with 2% fetal calf serum for 1 h at +4°C. For co-staining with intracellular markers, cells were then fixed with Fixation Buffer (00-8222-49, eBiosciences) for 30 min at +4°C and incubated with primary and secondary antibodies, as below, omitting methanol treatment.

For intracellular flow cytometry, cells were harvested using TrypLe Select (Gibco Life Technologies), washed and incubated with Fixation Buffer (00-8222-49, eBiosciences) for 30 min at +4°C. After washing with Permeabilization Buffer (00-8333-56, eBiosciences), cells were treated with 90% methanol for 30 min on ice, followed by three rounds of washing. Incubations with primary and secondary antibodies (electronic supplementary material, table S2) diluted with 5% donkey serum (Sigma-Aldrich) in Permeabilization Buffer were for 1 h at +4°C. Analysis was performed on a BD Fortessa LSR using FlowJo software.

### (f) Immunostaining

Cells were fixed with 4% buffered formaldehyde for 15 min at room temperature, permeabilized with 0.5% Triton X-100 in PBS for 10 min and blocked with 3% bovine serum albumin (BSA) and 0.1% Tween-20 in PBS for 30 min at room temperature. Incubation with primary antibodies (electronic supplementary material, table S3) diluted in blocking solution was carried out overnight at +4°C, then secondary antibodies were added for 1 h at room temperature. Cells were counterstained with DAPI and slides mounted with Prolong Diamond Antifade Mountant (Life Technologies).

### (g) Kidney capsule engraftment

PSC (H9, Shef6, AdiPS, FiPS) were differentiated to PDX1-expressing progenitors according to Kroon *et al*. [3]. The cells were gently scraped and washed with DMEM/F12 containing 0.1% BSA. After centrifugation cell pellets were transplanted under kidney capsules of NOD/SCID mice (9–12-week-old males; Charles River) using a glass micropipette (0.5–1 × 10^7^ cells per mouse).

### (h) C-peptide assay

Glucose-stimulated human C-peptide secretion was assayed by collecting blood samples from mice after overnight fast (16 h) and 30 min following intraperitoneal administration of d-(+)-Glucose (2 g/kg body weight; 30% solution; Sigma). Plasma was obtained after centrifugation of the blood samples (1600*g* for 10 min at 4°C) and human C-peptide levels were measured using an electrochemiluminescence assay (Meso Scale Discovery). Each assay contained a series of standards with concentrations of 0, 7.6, 22.8, 68, 204, 611, 1833, 5500 and 16 500 pmol l^−1^. The lower limit of detection (LLOD) was determined by the software analysing signals across the three lower concentration standards.

### (i) Immunohistological analysis

Recovered grafts and cell aggregates cultured on membrane at stage 7 of differentiation were rinsed in PBS and fixed for 1 hour in 4% paraformaldehyde. Following fixation, grafts were rinsed three times in PBS and incubated overnight at 4°C in 30% sucrose solution. The samples were then frozen in OCT solution and stored at −80°C before being cryosectioned at 7 µm thickness. Sections were rinsed in PBS for 5 min and blocked with an appropriate serum (5%) for 1 h. Primary antibodies at the appropriate dilution were added for 2 h at room temperature, followed by washing in PBS and incubation with secondary antibodies for 45 min at room temperature. Sections were then washed and mounted in Vectashield mounting medium. Sections were visualized using a Zeiss ApoTome fluorescence microscope.

## 3. Results

### (a) *In vivo* maturation of pancreatic progenitors generated from PSC

In order to assess the propensity of multiple PSC lines to form insulin-producing cells *in vivo*, we first used a seminal method for generation of pancreatic progenitors capable of maturation to glucose-responsive cells *in vivo* [3]. This method was originally established using two proprietary hESC cell lines CyT49 and CyT203. We applied the method to two well characterised hESC lines, H9 and Shef6, and two hiPSC lines and grafted differentiated cell populations under the kidney capsules of NOD/SCID immunodeficient mice.

**Figure 1.**
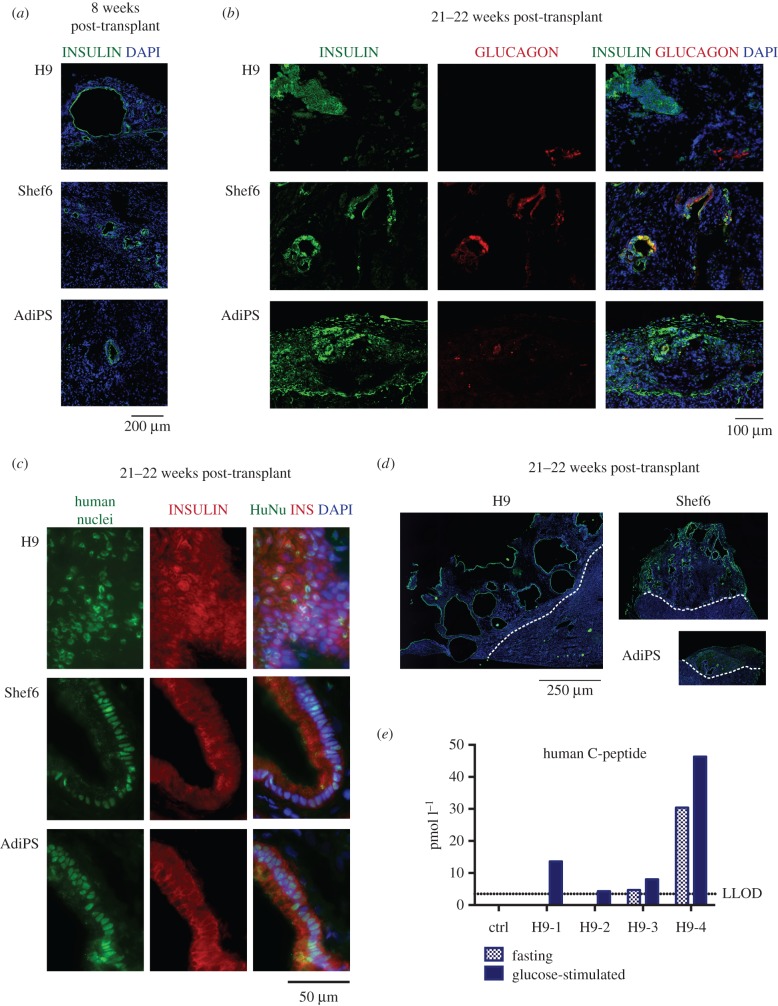
*In vivo* maturation of presumptive pancreatic endoderm generated from PSC according to Kroon *et al*. [3]. (*a*) Immunostaining for insulin of grafts eight weeks post-transplant derived from differentiated H9, Shef9 and AdiPS. (*b*) Immunostaining for insulin and glucagon, or (*c*) human nuclei of grafts 21–22 weeks post-transplant. (*d*) Immunostaining for insulin of grafts obtained 21–22 weeks after transplanting; representative images showing transversal sections of whole grafts. The images are tiled from multiple fields of view, scale bar for all 250 µm. (*e*) Glucose-induced C-peptide release in mice engrafted with H9-generated cells. Dotted line indicates lower limit of detection (3.48 pmol l^−1^), as defined by standards measurements.

We confirmed engraftment in all transplanted mice (table 3). In some cases tumour formation was observed with the highest incidence rate in mice engrafted with cells differentiated from FiPS. The non-tumourous grafts were analysed histologically at different time points: eight weeks, 13–15 weeks and 21–22 weeks after transplantation. A low proportion of insulin-expressing cells was detected as early as eight weeks after grafting (figure 1*a*). The number of insulin-positive cells gradually increased over time. Only a low number of cells expressed glucagon (figure 1*b*). We detected a proportion of polyhormonal INS^+^GCG^+^ cells in the Shef6-derived transplants whereas grafts recovered from H9 and AdiPS-transplanted mice contained monohormonal cells expressing either insulin or glucagon.

**Table 3.** Grafts of PSC-derived pancreatic progenitors generated according to Kroon *et al.* [3] in NOD SCID mice.

cell line	total number of transplanted mice	maximum time of follow-up (weeks)	incidence of tumours, detected/analysed (% from grafts)	number of non-tumourous grafts, detected/total analysed	mice with detectable GSIS, detected/analysed after 21–22 weeks
H9	8	21	0/8	8	4/4
Shef6	8	21	1/8 (12.5%)	7/8	0/4
AdiPS	8	22	0/8	8	0/3
FiPS	8	19	3/8 (37.5%)	5/8	n.a.

The grafts were composed mostly of cells of human origin as shown by immunostaining for human nuclei (figure 1*c*). The size of the grafts and frequency of insulin-positive cells were highly variable (figure 1*d*). The largest grafts were formed from H9 derivatives. However, these contained the lowest proportion of insulin-positive cells and displayed a number of other uncharacterised cell types. The grafts generated by all cell lines often had a cystic morphology (*n* = 17, out of 32), with insulin-positive cells lining the cavities, and not displaying the structure of pancreatic islets.

To assess functionality of endocrine cells in the grafts, we tested glucose-stimulated C-peptide secretion at different time points. We could reliably detect human C-peptide only in mice grafted with H9-derived cells after 21 weeks post-transplant (table 3, figure 1*e*). Two out of four mice had a low but detectable fasting level of C-peptide (4.7 and 30.4 pM; LLOD of the assay was 3.48 pM). Administration of glucose stimulated C-peptide secretion in all four of these mice, albeit at a low level.

We conclude that differentiated populations derived from four hESC and hiPSC lines using this protocol could generate low numbers of insulin-expressing cells *in vivo*, but these did not exhibit the morphological features of islets and showed no or low glucose responsiveness. Furthermore, the incidence of teratomas points to persistence of undifferentiated PSC in some cases.

### (b) Definitive endoderm differentiation *in vitro*

To assess whether the PSC used in our study may have some fundamental impairment in capacity to produce cells of pancreatic lineage, we systematically evaluated protocols for PSC differentiation to DE and subsequently to pancreatic progenitors.

The efficiency of DE formation was evaluated by flow cytometry for co-expression of the surface markers CD117 and CXCR4. A key transcription factor marker of DE, SOX17 [33], was present in almost all CD117^+^ CXCR4^+^ cells (electronic supplementary material, figure S1), confirming that co-expression of CD117 and CXCR4 is a reliable readout for quantification of DE production, as previously proposed [5,34–36].

**Figure 2.**
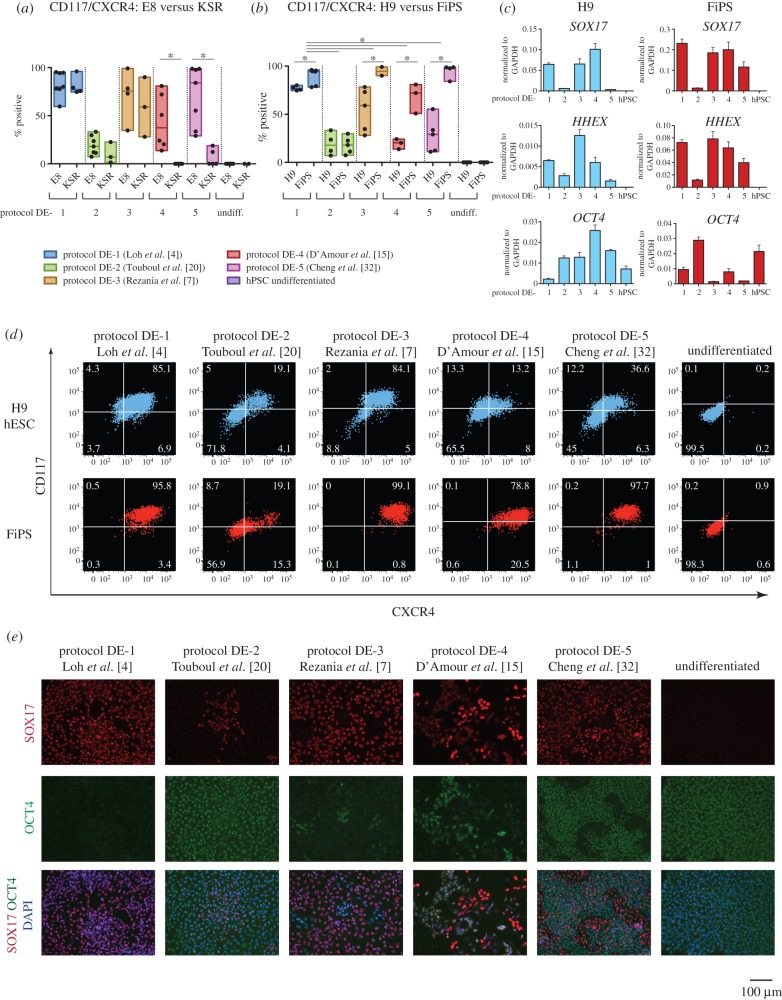
PSC differentiation to definitive endoderm. (*a*) PSC cultured in E8 or KSR/MEF conditions were differentiated using the five protocols and assayed by flow cytometry for CD117/CXCR4. Conditions resulting in no surviving cells were assigned 0% efficiency (protocols DE-4 and DE-5). Box plots show the results of independent biological replicates, individual values are indicated as dots. *p-value < 0.05. (*b*) Efficiency of DE differentiation was compared between H9 and FiPS cells. *p-value < 0.05. (*c*) Gene expression in H9 and FiPS-derived DE cells. A representative result of one experiment is shown. (*d*) Representative flow cytometry results for CD117 and CXCR4 expression in H9 and FiPS differentiated into DE using the five protocols. (*e*) Immunofluorescent staining for SOX17 and OCT4 in H9 differentiated to DE.

Standard protocols for PSC maintenance employ KSR and MEF. However, more defined culture conditions such as E8 medium have been recently developed. We therefore assessed the efficiency of DE differentiation from PSC expanded using KSR/MEF or E8 culture. We examined five published protocols for DE induction (summarized in table 1). We found that protocols DE-4 and DE-5 were poorly compatible with cells expanded in KSR/MEF, often resulting in complete cell death (8/10 experiments). The efficiencies of protocols DE-1, DE-2 and DE-3 were similar for KSR/MEF or E8 cultures (figure 2*a*, electronic supplementary material, table S5). Therefore, we used PSC maintained in both conditions for further analysis of DE induction using protocols DE-1, DE-2 and DE-3, and only E8-cultured PSC for protocols DE-4 and DE-5. We compared potential of hESC (H9) and hiPSC (FiPS) to generate DE using all five protocols. In all conditions except for DE-2, FiPS reproducibly showed rather higher efficiency of DE production than H9 (figure 2*b,d*, electronic supplementary material, table S6). Variation in lineage propensity among PSC lines is well documented [5,15]. We evaluated efficiency among the five chosen protocols for DE induction using combined results from H9 and FiPS differentiation. Protocol DE-1 was significantly more efficient relative to all the others, *p*-value < 0.05 (figure 2*b*,*d*, electronic supplementary material, table S7). Moreover, this protocol showed less difference between H9 and FiPS (median efficiencies 76.6% versus 94.5%, respectively) compared with most other conditions.

Flow cytometry results were validated by immunostaining of differentiated H9 hESC (figure 2*e*). We noted that the cell populations derived from the less efficient conditions (protocols DE-2, DE-4 and DE-5) still retained a large proportion of OCT4-highly positive cells, sometimes showing co-expression with a low level of SOX17. These cells might be primitive streak-like cells in process of specification to DE or may be mis-specified cells. Residual *OCT4* expression was confirmed at the mRNA level by qRT-PCR (figure 2*c*). We also observed that cells differentiated using protocol DE-4 reproducibly contained only a relatively small proportion of SOX17-positive cells, but that these cells exhibited intense staining. The relatively high expression of *SOX17* and *HHEX* mRNA at the population level (figure 2*c*) is therefore somewhat misleading for this protocol. This observation underlines that differentiation efficiency must be evaluated by protein expression at the cellular level and not solely by qRT-PCR.

We conclude that protocol DE-1 (performed according to Loh *et al*. [4]) has a higher efficiency and reproducibility of DE generation compared with the other protocols tested.

### (c) Generation of PDX1^+^ progenitors

We next sought to compare and evaluate existing methods for generation of early pancreatic progenitors from PSC-derived DE. We generated DE cells using the DE-1 protocol as above and then applied a range of conditions reported to specify DE to primitive gut tube and further to PDX1-expressing presumptive pancreatic endoderm (table 2). Protocols P-1 [3] and P-2 [5] have previously been reported to generate PDX1-positive progenitors that could differentiate further into polyhormonal endocrine cells expressing both glucagon and insulin *in vitro*. Additionally, cells produced using protocol P-1 were shown to mature to monohormonal glucose-responsive cells *in vivo* [3]. Protocol P-3 was reported as a method for production of PDX1-positive cells without further evaluation [4]. Protocol P-4 was used in two recent reports by Rezania *et al*. [7] and Pagliuca *et al*. [6], to obtain PDX1-expressing progenitors (called stage 3 cells, S3). In these two studies, differing additional steps were applied after P-4 to enrich for PDX1^+^ NKX6.1^+^ double-positive cells, called stage 4 cells, S4 (protocols P-5 and P-6 in our experimental set-up). Those progenitors displayed capacity to differentiate to monohormonal insulin-expressing cells both *in vitro* and *in vivo* [6,7].

**Figure 3.**
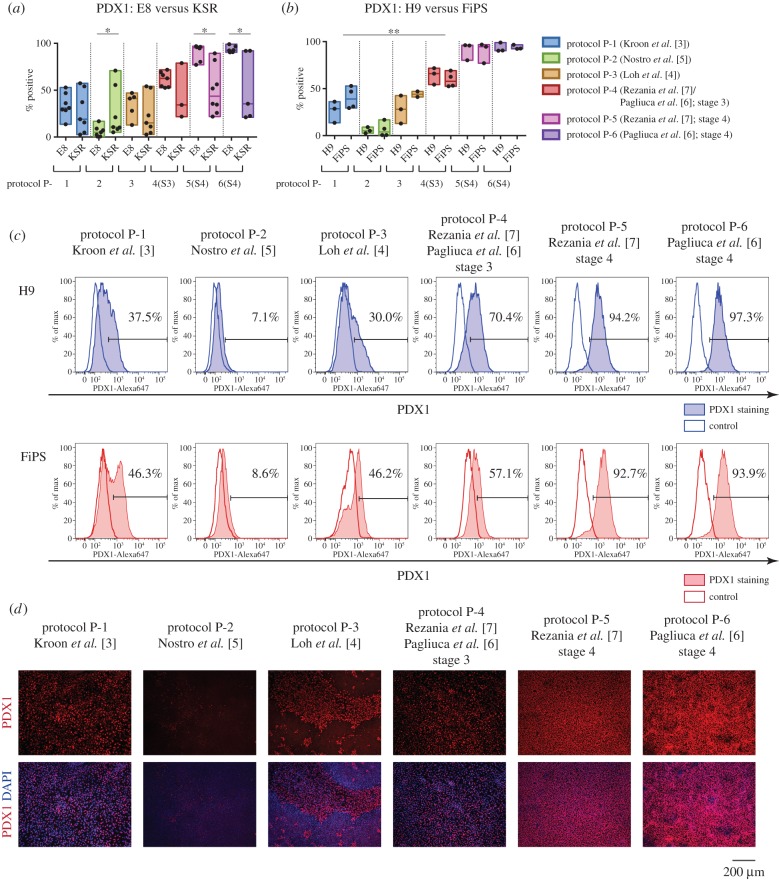
Differentiation from DE to PDX1^+^ presumptive pancreatic endoderm. (*a*) DE cells generated from PSC using protocol DE-1 were differentiated further using six protocols. Efficiency was quantified by flow cytometry for PDX1. The results for independent biological replicates are summarized in box plots and individual values are shown as dots. *p-value < 0.05. (*b*) Efficiency of differentiation to PDX1^+^ progenitors was compared between E8-cultured H9 and FiPS cells. **p-value < 0.01. (*c*) Representative flow cytometry results for PDX1 expression in H9 and FiPS cells differentiated using six protocols. (*d*) Immunofluorescent staining for PDX1 of populations derived from H9 using different protocols.

We first compared the capacity of PSC cultured in KSR/MEF or E8 to differentiate beyond DE to PDX1-expressing cells. We quantified outcomes by intracellular flow cytometry. Protocols P-5 and P-6 were more efficient using E8-cultured cells, and protocol P-2 was more efficient starting from cells in KSR/MEF (figure 3*a*, electronic supplementary material, table S8). However, the outcome for KSR-cultured cells was more variable for each protocol than for cells maintained in E8, as indicated by the coefficient of variation. Therefore, we decided to use only cells cultured in E8 for further comparisons between the protocols.

We examined the potential of H9 and FiPS to generate PDX1^+^ cells using the six protocols. Interestingly, there was no significant difference in production of PDX1-positive cells between H9 and FiPS cells using all tested methods (figure 3*b*, electronic supplementary material, table S9). By analysing combined results of H9 and FiPS differentiation, we found the lowest proportion of PDX1-expressing cells after protocol P-2 (median 5.1%), and higher levels after protocols P-1 and P-3 (median 31.0% and 41.1%, respectively), although protocol P-3 often resulted in high levels of cell death for both cell lines. Protocol P-4 (stage 3 according to [6,7]) yielded significantly greater numbers of PDX1-positive cells (median 62.6%) compared with the other three conditions. PDX1 was further elevated applying protocols P-5 and P-6 (stage 4), resulting in conversion of the vast majority of cells to PDX1-positive progenitors (95.3% and 92.6%, respectively) (figure 3*b*,*c*,*d*, electronic supplementary material, table S10).

**Figure 4.**
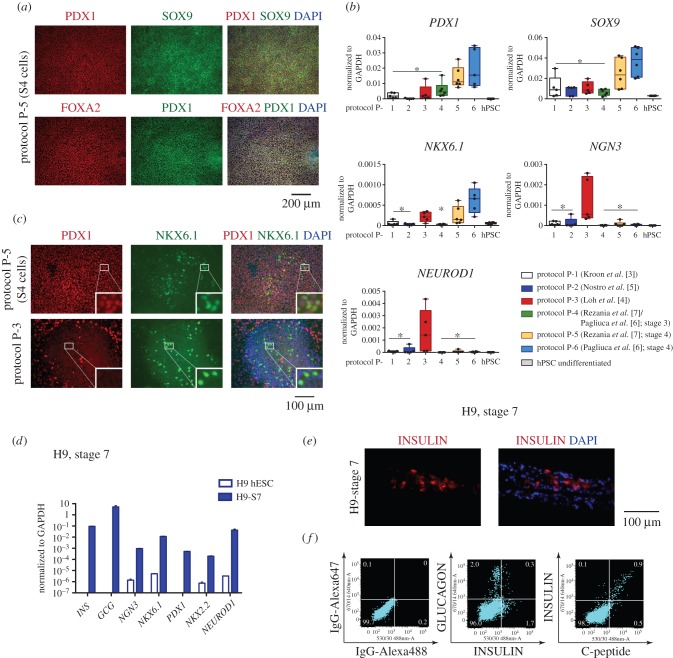
Characterisation of PSC-derived presumptive pancreatic endoderm. (*a*) Immunostaining for PDX1, FOXA2 and SOX9 in differentiated cells derived from H9 cells using protocol 5 (S4 stage). (*b*) Gene expression in differentiated cells derived from PSC using the six protocols. Whisker plots summarize the results for independent biological replicates, and individual values are shown as dots. (*c*) Immunofluorescent staining for PDX1 and NKX6.1. A proportion of cells generated by protocol 5 (S4 stage) co-expressed PDX1 and NKX6.1, whereas the NKX6.1^+^ cells derived using protocol 3 did not have detectable PDX1. Insets show higher magnification of regions marked with a white square. (*d*) Gene expression after further differentiation of PDX1^+^ cells generated from H9 using protocol 5 (S7 stage, [7]). (*e*) Immunostaining for insulin and (*f*) flow cytometry results for insulin (rabbit anti-human antibody) in combination with glucagon, and insulin (mouse anti-human antibody) with C-peptide in S7 stage cells derived from H9.

Progenitors generated from PSC co-expressed PDX1, SOX9 and FOXA2 (figure 4*a*). We also confirmed upregulation of NKX6.1 in the stage 4 cells after protocols P-5 and P-6, and emergence of PDX1^+^ NKX6.1^+^ double-positive cells (figure 4*b*,*c*). Protocol P-3 also reproducibly resulted in an elevated level of NKX6.1, but the protein was present only in cells with low or undetectable PDX1 (figure 4*c*). This observation suggests that these cells either belong to another lineage, or represent more advanced differentiated endocrine cells. The latter explanation would be consistent with the expression of NGN3 and NEUROD1 mRNA after protocol P-3 (figure 4*b*).

Finally, we assessed whether PDX1^+^ progenitors from protocol P-5 could be further differentiated to insulin-positive cells using a recently described transwell culture system [7]. Expression of markers characteristic for hormone-expressing cells was shown by qPCR and antibody staining (figure 4*d–f*) and insulin synthesis was confirmed by flow cytometry for C-peptide. Glucagon-positive cells were also present but importantly most of the insulin-expressing cells were monohormonal. However, the frequency of insulin-positive cells was only 1%, lower than reported previously for a single hESC cell line [7], and prohibited reliable assay of glucose sensitivity or engraftment.

## 4. Discussion

Efficient generation of pancreatic lineage derivatives is important for research in human beta cell biology, drug testing, immunological studies of the aetiology of type I diabetes, and development of cell replacement therapy. Multiple protocols have been developed to use PSC as a source of pancreatic lineage *in vitro* [4–11] and *in vivo* [3,6,7], however, those have generally been validated with a single or very limited number of cell lines. Furthermore, very few studies have examined the capacity of hiPSC for *in vivo* maturation [37].

In this study, we first assessed pancreatic progenitors derived from two hESC and two hiPSC lines using a previously published method [3] for the ability to produce functional beta cells *in vivo*. For engraftment we used differentiating cells at the onset of PDX1 expression. PDX1 is the earliest marker for pancreatic progenitors that give rise to all pancreatic lineages [24,25]. However, other cell types in stomach and duodenum also express PDX1 during development [26,27], and may be present in the differentiating cultures. PDX1-expressing cell populations produced from all PSC lines were able to produce some insulin-expressing cells *in vivo*. Notably, most grafts contained large cysts lined with insulin-positive cells, which did not form islet-like structures. Interestingly, in a later study the authors of the original method revealed that approximately one half of grafts exhibited such morphology with detectable glucose-stimulated insulin secretion (GSIS) in those mice [38]. Our observation is therefore not inconsistent.

We detected glucose-induced human C-peptide release only in mice that received H9-derived grafts. Furthermore, this response was very low. This result contrasts with the high C-peptide level observed in most transplanted mice in the original report [3,38]. The other difference we observed was the appearance of polyhormonal cells co-expressing insulin and glucagon in grafts from Shef6 hESC, which persisted at 21 weeks after transplant. It should be noted that we transplanted PSC-generated early pancreatic progenitors (PDX1^+^) whereas Kroon *et al*. used cells at a later stage of specification (NKX6.1^+^ NGN3^+^ NKX2.2^+^), which could potentially affect the outcome. In their original study the protocol was validated using two in-house hESC lines, CyT49 and CyT203. However, others have also reported lack of functional beta cells in transplants from hiPSC derivatives following this approach [37]. Of additional note, we observed incidences of tumour formation from some of the transplanted cells. This indicates that cell populations derived from PSC after differentiation using this protocol may still contain pluripotent cells.

We investigated whether the lack of functional beta cells in the grafts reflected intrinsic differentiation deficiencies in the PSC used in our study. For this, we systematically evaluated several existing methods to generate DE and subsequently PDX1-expressing pancreatic progenitors from PSC. We assessed efficiencies depending on the conditions of PSC expansion, cell line and protocol. PSC are commonly maintained using fibroblast feeder layers and KSR [29], although several more defined culture conditions have now been developed (reviewed in [39]). We compared the outcome of differentiation of PSC grown in KSR/MEF and E8. The potential to generate DE was not dependent on starting conditions. However, the following steps of differentiation to pancreatic lineage were more reproducible and robust using PSC expanded in E8 rather than in KSR/MEF. These data suggest that the propensity of PSC for later steps of differentiation may be influenced by their prior expansion conditions. Alternatively, persistence of MEFs may compromise pancreatic differentiation after DE specification. In general terms, the superior performance of cells maintained in defined E8 medium bodes well for translation to protocols for good laboratory practice (GLP) and good manufacturing practice (GMP).

We examined different PSC lines for their capacity to produce PDX1^+^ progenitors. Perhaps unsurprisingly, the early steps of differentiation were variable among lines, as observed in previous studies [5,15]. By contrast, the outcome of the later specification at the PDX1^+^ stage was not significantly different among lines, if differentiation was performed under optimal conditions (i.e. feeder-free expansion and efficient induction to DE).

Five protocols to induce DE from PSC and six protocols to differentiate DE further to presumptive PDX1-expressing pancreatic endoderm were examined. We found that the protocol reported by Loh *et al*. [4] was the most efficient and robust for DE derivation among all lines that we tested. The specific features of this method include use of FGF2 and temporal modulation of BMP signalling (slight activation for one day and inhibition later on). Some PSC lines could generate DE with high efficiency using protocols that do not involve those factors, however. Therefore, DE induction does not absolutely require exogenous FGFs and BMP inhibition, but these signals may improve the outcome for particular cell lines.

Among the protocols for pancreatic progenitor generation, the recently reported methods by Rezania *et al*. [7] and Pagliuca *et al*. [6] were the most efficient and reproducible for the four PSC lines tested. At stage 3 of this protocol cells expressed PDX1 with a higher frequency compared with the other approaches, and the level and proportion were further increased during stage 4, resulting in cell populations containing more than 90% PDX1-expressing progenitors. Furthermore, we could further differentiate progenitors after protocol P-5 to monohormonal insulin-expressing cells *in vitro*. In the original report, insulin-expressing cells derived using this approach could restore glucose levels in diabetic mice after transplantation [7]. However, in our hands the yield was very low, around 1%, suggesting further optimization is required for scalable production.

In summary, among published protocols we identified conditions for efficient and robust generation of pancreatic progenitors, including endocrine progenitors, from different PSC lines. However, even using the optimal conditions defined here (stage 4 cells, protocol P-5) we have observed that teratomas frequently formed after grafting to immunocompromised mice (data not shown). We therefore suggest that an attractive alternative for cell-based therapeutic approaches to type I diabetes would be to define conditions that enable capture and stable expansion of pure pancreatic progenitors.

## Supplementary Material

Supplementary Information
