# Supplementary material for: Towards consistent generation of pancreatic lineage progenitors from human pluripotent stem cells
Source: Philos Trans R Soc Lond B Biol Sci. 2015 Oct 19;370(1680):20140365. doi: 10.1098/rstb.2014.0365 (PMC4633994; doi:10.1098/rstb.2014.0365)
Supplement: Supplementary Information [file rstb20140365supp1.pdf]

Consistent Generation of Pancreatic Lineage Progenitors from Human Pluripotent Stem Cells

Supplementary Information

Supplementary Experimental Procedures
Supplementary Figure 1
Supplementary Tables 1-10

Supplementary Experimental Procedures

PSC culture

Undifferentiated PSCs were maintained in two culture conditions: on feeder layers of γ-irradiated mouse embryonic fibroblasts (MEF) or on Matrigel. For culture on MEF (KSR/MEF conditions), the growth medium comprised DMEM-F12 (Sigma-Aldrich), 20% KnockOut Serum Replacement, KSR, 0.1mM b-mercaptoethanol, 2mM L-Glutamine (all Gibco Life Technologies), 1x non-essential aminoacids (Sigma), 10ng/ml FGF-2 (produced in house). Cells were routinely passaged at 1 in 4 dilution every 5-7 days using 1mg/ml dispase (Roche). 10μM Rho-associated kinase inhibitor (ROCKi, Y-27632, Calbiochem) was added for 24 hours after replating.
On Matrigel-coated dishes (Corning) cells were cultured in Essential 8 medium, E8 (Gibco Life Technologies). At 70-80% of confluency cells were dissociated using 0.5mM EDTA and plated at 1:10-1:4 dilution.
Cells were cultured in 5% CO_2, 5% O_2 and 90% N_2 in a humidified incubator at 37°C.

Definitive endoderm differentiation

Five protocols were applied to differentiate PSC to DE. To determine optimal starting cell density, each protocol was tested with PSC cultures at 20%, 50% and 80% confluency. The media were changed daily.
Growth factors and chemical inhibitors and activators are listed in Suppl. Table 4.

Protocol DE-1 (Loh et al., 2014)
Differentiation was initiated with cells at 20-30% confluency. Higher density significantly reduced the efficiency of differentiation.

Basal medium CDM2 consisted of a 1:1 mixture IMDM and F12 (Gibco Life Technologies) 2 mM L-Glutamine, 0.1% BSA (A7906, Sigma), 1% Chemically defined lipid concentrate (11905-031, Gibco Life Technologies), 15μg/ml transferrin (T8158, Sigma), 450μM monothioglycerole (Sigma), 0.7μg/ml insulin.

On day 1 of differentiation, cells were exposed to CDM2 supplemented with 100ng/ml Activin A, 100nM PI-103, 3μM CHIR99021, 10ng/ml FGF2, 3ng/ml BMP4. On day 2 and 3 of differentiation, the medium was CDM2 supplemented with 100ng/ml Activin A, 100nM PI-103, 20ng/ml FGF2, 250nM LDN193189/DM3189.

Protocol DE-2 (Touboul et al., 2010), with slight modification
Differentiation was initiated with cells at 20-30% confluency.
Basal medium (CDM) comprised a 1:1 mixture of IMDM and F12 supplemented with 2mM L-Glutamine, 0.5% BSA, 1% Chemically defined lipid concentrate, 15μg/ml transferrin, 450μM monothioglycerole and 7μg/ml insulin. Cells were differentiated in CDM with 100nM PI-103, 100ng/ml Activin A, 20ng/ml FGF2 and 10ng/ml BMP4, for 3 days.
PI-103 inhibitor was used instead of Ly294002 applied in the original method.

Protocol DE-3 (Rezania et al., 2014)
Differentiation was initiated with the cells at 20-30% of confluency.

© The Authors under the terms of the Creative Commons Attribution License http://creativecommons.org/licenses/by/3.0/, which permits unrestricted use, provided the original author and source are credited.

The basal medium for differentiation consisted of MCDB131 (Gibco Life Technologies) supplemented with 1.5g/L NaHCO3, 2mM L-Glutamine, 10mM Glucose (Sigma), 0.5% BSA. The cells were exposed to 100ng/ml GDF8 and 3μM CHIR99021 for 1 day, then to 100ng/ml GDF8 and 0.3μM CHIR99021 for 1 day, then to 100ng/ml GDF8 for 1 day.

Protocol DE-4 (D'Amour et al., 2005)
Differentiation was initiated with cells at 80-90% confluency, the conditions of differentiation were toxic for cells at lower density. The basal medium for the protocol was RPMI (Gibco Life Technologies) with 2mM L-Glutamine. Cells were exposed to 100ng/ml Activin A and 25ng/ml Wnt3a for 1 day, followed by 2 days with 0.2% FBS and 100ng/ml Activin A.

Protocol DE-5 (Cheng et al., 2012)
Differentiation was initiated with cells at 80-90% confluency, the conditions of differentiation were toxic for the cells at lower density.
The basal media for the protocol were RPMI+2mM L-Glutamine or SFD (IMDM:Ham F12 in 3:1 ratio, 0.1% BSA, 1% B27 without vitamin A (12587-010, Gibco Life Technologies), 0.5% N2 (17502-048, Gibco Life Technologies), 50μM ascorbic acid, 450μM monothioglycerole). For the first day of the differentiation cells received medium comprised of 90% RPMI, 10% SFD, 40ng/ml Wnt3a, 100ng/ml Activin A. The following two days the medium was RPMI with 0.5ng/ml BMP4, 10ng/ml FGF2, 100ng/ml Activin A, 10ng/ml VEGF. On days 4 and 5 the medium was SFD supplemented with 0.5ng/ml BMP4, 10ng/ml FGF2, 100ng/ml Activin A, 10ng/ml VEGF.

Pancreatic differentiation

For pancreatic induction PSCs were first differentiated to DE according to the protocol DE-1 as described above. Six different protocols were applied to derive PDX1$^+$ presumptive pancreatic endoderm.

Protocol P-1 (Kroon et al., 2008)
Stage 2: RPMI supplemented with 2mM L-Glutamine, 2% FCS and 50ng/ml FGF7, for 3 days.
Stage 3: DMEM supplemented with 1% B27 without vitamin A, 2μM all-trans retinoic acid (ATRA, R2625, Sigma), 250nM SANT-1, 250nM LDN193189/DM3189, for 3 days.

Protocol P-2 (Nostro et al., 2011)
Stage 2: SFD (as above) supplemented with 3ng/ml Wnt3a, 50ng/ml FGF10, 250nM LDN193189/DM3189, for 3 days.
Stage 3: DMEM supplemented with 1% B27 without vitamin A, 2μM ATRA, 250nM SANT-1, 250nM LDN193189/DM3189, 50ng/ml FGF10, for 3 days.

Protocol P-3 (Loh et al., 2014)
Stage 2: SFD (as above) supplemented with 250nM LDN193189/DM3189, 4μM IWP2, 500nM PD0325901, 2μM ATRA, for 1 day.
Stage 3: DMEM supplemented with 1% B27 without vitamin A, 2μM ATRA, 250nM SANT-1, 250nM LDN193189/DM3189, 500nM PD0325901, for 3 days.

Protocol P-4 (Rezania et al., 2014)
Stage 2: basal medium was MCDB131 with 1.5g/L NaHCO3, 2mM L-Glutamine, 10mM Glucose and 0.5% BSA. The cells were cultured in the presence of 250μM ascorbic acid (Sigma) and 50ng/ml FGF7, for 2 days.
Stage 3: basal medium was MCDB131 with 2.5g/L NaHCO3, 2mM L-Glutamine, 10mM Glucose and 2% BSA. The following components were added: 1:200 ITS-X (51500-056, Gibco Life Technologies), 250μM ascorbic acid, 50ng/ml FGF7, 250nM SANT-1, 1μM ATRA, 100nM LDN193189/DM3189, 200nM PKC activator TPB ((2S,5S)-(E,E)-8-(5-(4-(Trifluoromethyl)phenyl)-2,4-pentadienoylamino)benzolactam), for 2 days.

Protocol P-5 (Rezania et al., 2014)
Cells were differentiated according to the protocol 4, then an additional stage of treatment was applied.

© The Authors under the terms of the Creative Commons Attribution License http://creativecommons.org/licenses/by/3.0/, which permits unrestricted use, provided the original author and source are credited.

Stage 4: basal medium was MCDB131 with 2.5g/L NaHCO3, 2mM L-Glutamine, 10mM Glucose and 2% BSA. The following components were added: 1:200 ITS-X, 250μM ascorbic acid, 2ng/ml FGF7, 250nM SANT-1, 0.1μM ATRA, 200nM LDN193189/DM3189, 100nM PKC activator TPB, for 3 days.

Protocol P-6 (Pagliuca et al., 2014), with slight modification
Cells were differentiated according to the protocol 4, then an additional stage of treatment was applied.
Stage 4: basal medium was MCDB131 with 2.5g/L NaHCO3, 2mM L-Glutamine, 10mM Glucose and 2% BSA. The following components were added: 1:200 ITS-X, 250μM ascorbic acid, 50ng/ml FGF7, 250nM SANT-1, 100nM ATRA, for 5 days.
PKC activator TPB was used instead of originally reported phorbol 12,13-dibutyrate (PdBU).

Differentiation into insulin-expressing cells
Progenitors derived after protocol P-5 were differentiated according to the original report (Rezania et al., 2014). In brief, the stage 4 cells were incubated with ROCKi for 1 hour, then dissociated and plated on membrane in transwells (CLS3414-24EA, Sigma) as aggregates of $5x10^5$ cells. At all stages basal medium was MCDB131 with 1.5g/L NaHCO3, L-Glutamine, 20mM Glucose, 2% BSA.
Stage 5: the following supplements were added for 3 days: 1:200 ITS-X, 250nM SANT-1, 50nM ATRA, 100nM LDN193189/DM3189, 1μM 3,3′,5-Triiodo-L-thyronine sodium salt, T3 (T6397, Sigma), 10μM Alk5 inhibitor II, 10μM ZnSO4 (Z0251, Sigma), 10μg/ml heparin (H3149, Sigma).
Stage 6-1: for 7 days the medium was supplemented with 1:200 ITS-X, 100nM LDN193189/DM3189, 1μM T3, 10μM Alk5 inhibitor II, 10μM ZnSO4, 100nM γ-secretase inhibitor XX.
Stage 6-2: for the following 8 days the medium was supplemented with 1:200 ITS-X, 100nM LDN193189/DM3189, 1μM T3, 10μM Alk5 inhibitor II, 10μM ZnSO4, 10μg/ml heparin.
Stage 7: the following supplements were added for 15 days: 1:200 ITS-X, 1μM T3, 10μM Alk5 inhibitor II, 10μM ZnSO4, 1mM N-acetyl-cystein (A9165, Sigma), 10μM Trolox (648471, EMD Millipore), 2μM R428 inhibitor. During the last 8 days, 10μg/ml heparin was additionally applied.

© The Authors under the terms of the Creative Commons Attribution License http://creativecommons.org/licenses/by/3.0/, which permits unrestricted use, provided the original author and source are credited.

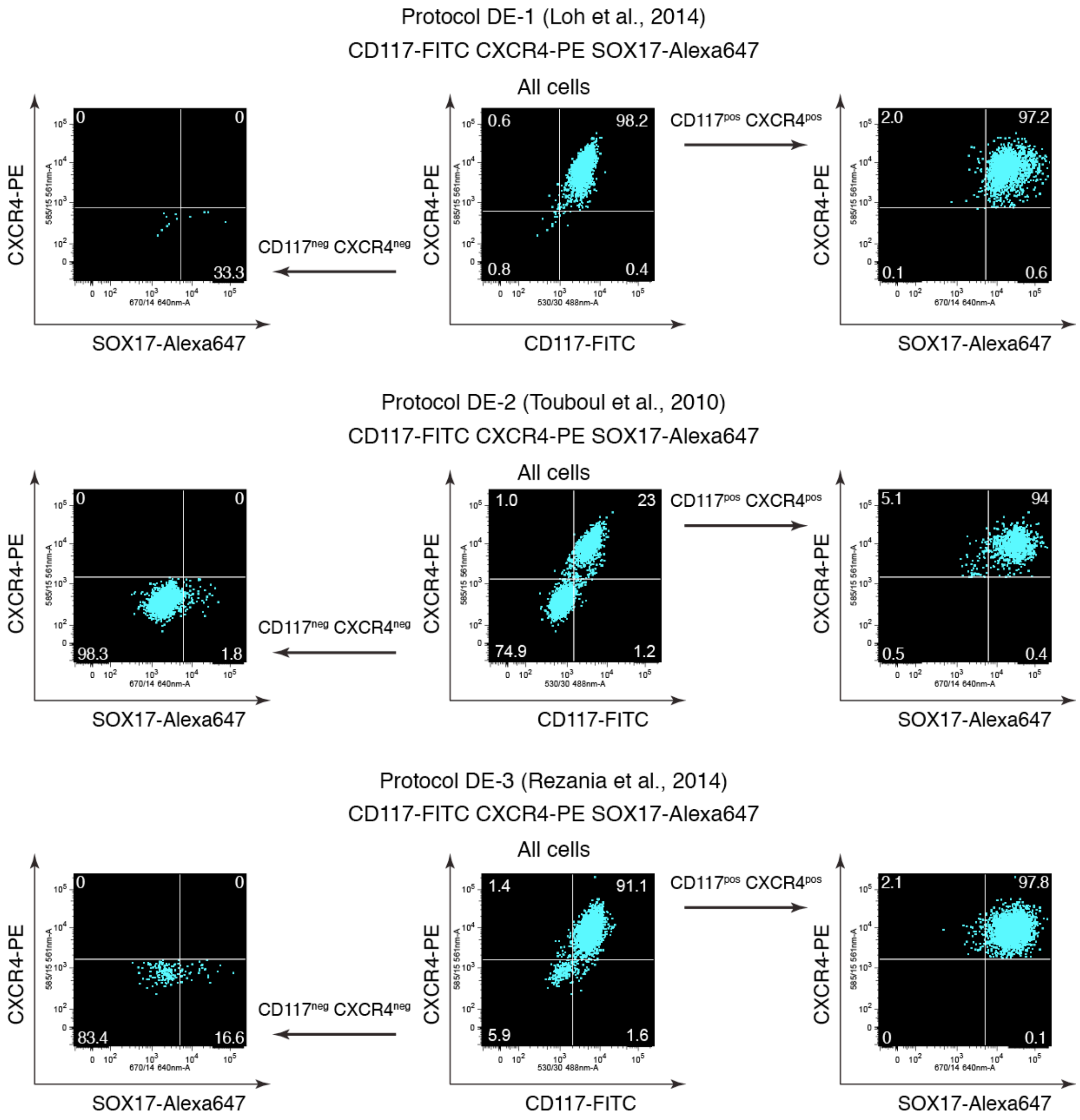

Supplementary Figure 1: Marker expression in PSC differentiated to DE. FiPS differentiated to DE using three protocols (DE-1, DE-2 and DE-3, Table 1) were simultaneously stained with antibodies for CD117-FITC / CXCR4-PE / SOX17-Alexa647. Middle panel shows that cells in differentiated populations were either double positive or double negative for CD117 and CXCR4. In addition, the majority of double negative cells did not express SOX17 (left panel), and most of double positive co-expressed SOX17 (right panel), thus corroborating DE specification.

© The Authors under the terms of the Creative Commons Attribution License http://creativecommons.org/licenses/by/3.0/, which permits unrestricted use, provided the original author and source are credited.

Supplementary Tables

mRNA	Forward (5'-3')	Reverse (5'-3')	Product size	UPL probe
OCT4	cttcgcaagccctcatttc	gagaaggcgaaatccgaag	88	60
SOX17	acgccgagttgagcaaga	tctgcctcctccacgaag	82	61
FOXA2	cgttccgggtctgaactg	accgctcccagcatacttt	77	68
HHEX	cggacggtgaacgactaca	agaaggggctccagagtagag	76	61
PDX1	aagctcacgcgtggaaag	ggccgtgagatgtacttgttg	145	78
SOX9	gtacccgcacttgcacaac	tcgctctcgttcagaagtctc	72	61
NKX6.1	cgttggggatgacagagagt	cgagtcctgcttcttcttgg	114	69
NGN3	cgctcattttaggcctcct	ccgggtagtgctaccattc	76	77
NEUROD1	aaagccctctgactgattgc	gaagttgccattgatgctga	77	30
NKX2.2	gaggaaggcaggggtcat	actcggagcaccctggat	91	23
INS	aggcttcttctacacacccaag	cacaatgccacgcttctg	139	16
GCG	gtacaaggcagctggcaac	tgggaagctgagaatgatctg	71	82

Supplementary Table 1: Primer sequences.

© The Authors under the terms of the Creative Commons Attribution License http://creativecommons.org/licenses/by/3.0/, which permits unrestricted use, provided the original author and source are credited.

Antibody	Cat. Number	Company	Dilution
FITC-conjugated mouse IgG1 anti-human CD117 (c-Kit), clone 104D2	11-1178-42	eBioscience	1:25
Polyclonal goat IgG anti-human SOX17	AF1924	RnD	1:1000
PE conjugated mouse IgG2a anti-human CD184 (CXCR4)	555974	BD Pharmingen	1:25
Polyclonal goat IgG anti-human PDX1	AF2419	RnD	1:1000
Mouse IgG1 anti-mouse/human PDX1 (clone 658A5)	562160	BD Pharmingen	1:50
Mouse IgG1 anti-human Insulin (clone K36AC10)	I2018	Sigma	1:1000
Alexa488-conjugated rabbit IgG anti-human/mouse/rat Insulin (clone C27C9)	9016S	Cell Signaling Technology	1:50
Polyclonal rabbit anti-human/mouse/rat C-peptide	4593S	Cell Signaling Technology	1:25
Mouse IgG1 anti-human Glucagon (clone K79bB10, ascites fluid)	G2654	Sigma	1:1000
Alexa647-conjugated donkey anti-goat IgG (H+L) secondary antibody	A21447	Molecular Probes, Life Technologies	1:1000
Alexa488-conjugated donkey anti-rabbit IgG (H+L)	A21206	Molecular Probes, Life Technologies	1:2000
Alexa647-conjugated donkey anti-mouse IgG (H+L)	A31571	Molecular Probes, Life Technologies	1:1000
Alexa488-conjugated donkey anti-mouse IgG (H+L) secondary antibody	A21202	Molecular Probes, Life Technologies	1:1000

Supplementary Table 2: List of antibodies used for flow cytometry.

© The Authors under the terms of the Creative Commons Attribution License http://creativecommons.org/licenses/by/3.0/, which permits unrestricted use, provided the original author and source are credited.

Antibody	Cat. number	Company	Dilution
Mouse IgG2b anti-human OCT3/4 (clone C-10)	sc-5279	Santa Cruz	1:100
Polyclonal goat IgG anti-human SOX17	AF1924	RnD	1:100
Polyclonal goat IgG anti-human PDX1	AF2419	RnD	1:50
Mouse IgG1 anti-mouse/human PDX1 (clone 658A5)	562160	BD Pharmingen	1:50
Mouse IgG1 anti-human/mouse SOX9 (clone GMPR9)	14-9765	eBioscience	1:100
Mouse IgG1 anti-rat/human NKX6.1 (clone F55A10)	F77A10	Developmental Studies Hybridoma Bank	1:10
Polyclonal goat IgG anti-human HNF-3b/FOXA2	AF2400	RnD	1:50
Polyclonal guinea pig anti-human Insulin	A0564	Dako	1:200 – 1:2000
Mouse IgG1 anti-human nuclei	MAB1281	Chemicon Millipore	1:200
Alexa555-conjugated donkey anti-goat IgG (H+L) secondary antibody	A21432	Molecular Probes, Life Technologies	1:2000
Alexa488-conjugated goat anti-guinea pig IgG (H+L) secondary antibody	A11073	Molecular Probes, Life Technologies	1:1000
Alexa488-conjugated donkey anti-mouse IgG (H+L) secondary antibody	A21202	Molecular Probes, Life Technologies	1:1000
Cy3-conjugated donkey anti-guinea pig IgG secondary antibody	AP193C	Chemicon Millipore	1:200

Supplementary Table 3: List of antibodies used for immunocytochemistry.

© The Authors under the terms of the Creative Commons Attribution License http://creativecommons.org/licenses/by/3.0/, which permits unrestricted use, provided the original author and source are credited.

Compound	Cat. number	Company
Activin A	Produced in house	
BMP4	120-05ET	Peprotech
GDF8	120-00	Peprotech
Wnt3a	5036-WN-010	RnD Bio-Techne
VEGF	100-20	Peprotech
FGF2	Produced in house	
FGF7	100-19	Peprotech
FGF10	100-26	Peprotech
ROCKi Y-27632<br><br>(Rho-associated kinase inhibitor)	688000	Calbiochem
PI-103<br><br>(PI3K inhibitor)	2930	Tocris Bio-Techne
LDN193189/DM3189<br><br>(BMP receptor ALK2/3/6 inhibitor)	1509	Axon
SANT-1<br><br>(Hedgehog inhibitor, Smoothened antagonist)	1974	Tocris Bio-Techne
IWP2<br><br>(inhibitor of Wnt processing and secretion)	3533	Tocris Bio-Techne
TPB, (2S,5S)-(E,E)-8-(5-(4-(Trifluoromethyl)phenyl)-2,4-pentadienoylamino)benzolactam<br><br>(PKC activator)	565740	EMD Millipore
ALK5 inhibitor II<br><br>(TGFb receptor ALK5 inhibitor)	ALX-270-445	Enzo Life Sciences
γ-secretase inhibitor XX	565789	EMD Millipore
R428<br><br>AXL inhibitor	S2841	SelleckBiochem

Supplementary Table 4: List of cytokines and chemical inhibitors/activators.

© The Authors under the terms of the Creative Commons Attribution License http://creativecommons.org/licenses/by/3.0/, which permits unrestricted use, provided the original author and source are credited.

Protocol	Reference	% CD117/CXCR4 positive after E8, median value	% CD117/CXCR4 positive after KSR, median value	*p*-value
1	Loh et al., 2014	80.0 (n=7)	77.3 (n=4)	0.42485
2	Touboul et al., 2010	18.0 (n=7)	7.1 (n=3)	0.11790
3	Rezania et al., 2014	75.65 (n=4)	59 (n=3)	0.29964
4	D'Amour et al., 2005	37.45 (n=6)	0* (n=5)	**0.00400**
5	Cheng et al., 2012	84.1 (n=7)	0* (n=5)	**0.00061**

* in 8/10 cases no cells survived

Supplementary Table 5: Definitive endoderm differentiation of PSCs cultured in E8 compared to KSR conditions. Numbers of data points are indicated for each median value. Significantly different comparisons are shown in bold.

© The Authors under the terms of the Creative Commons Attribution License http://creativecommons.org/licenses/by/3.0/, which permits unrestricted use, provided the original author and source are credited.

Protocol	Reference	H9<br><br>% CD117/CXCR4 positive,<br><br>median value	FiPS<br><br>% CD117/CXCR4 positive,<br><br>median value	*p*-value
1	Loh et al., 2014	76.6 (n=4)	94.5 (n=6)	**0.010904**
2	Touboul et al., 2010	17.8 (n=4)	18.0 (n=5)	0.434817
3	Rezania et al., 2014	59.0 (n=5)	94.55 (n=2)	**0.032831**
4	D'Amour et al., 2005	20.3 (n=3)	72.0 (n=3)	**0.003381**
5	Cheng et al., 2012	29.0 (n=5)	98.2 (n=4)	**0.00012**

Supplementary Table 6: Definitive endoderm differentiation of H9 compared to FiPS cells. Numbers of data points are indicated for each median value. Significantly different comparisons are shown in bold.

© The Authors under the terms of the Creative Commons Attribution License http://creativecommons.org/licenses/by/3.0/, which permits unrestricted use, provided the original author and source are credited.

	Reference	% CD117/CXCR4 positive (median)	*p*-value			
			Protocol 2	Protocol 3	Protocol 4	Protocol 5
			Touboul et al., 2010	Rezania et al., 2014	D'Amour et al., 2005	Cheng et al., 2012
1	Loh et al., 2014	79.55% (n=10)	**1.1x10E-11**	**0.029974**	**0.000395**	**0.021871**
2	Touboul et al., 2010	18.0% (n=9)		**0.000104**	**0.013386**	**0.003804**
3	Rezania et al., 2014	73.0% (n=7)			0.087033	0.317804
4	D'Amour et al., 2005	37.5% (n=6)				0.223785
5	Cheng et al., 2012	55.3% (n=9)				

Supplementary Table 7: Definitive endoderm differentiation of PSC using different methods. P-values are shown for each pairwise comparison. Numbers of data points are indicated for each median value. Significantly different comparisons are shown in bold.

© The Authors under the terms of the Creative Commons Attribution License http://creativecommons.org/licenses/by/3.0/, which permits unrestricted use, provided the original author and source are credited.

Protocol	Reference	% PDX1 positive after E8,<br><br>median value	% PDX1 positive after KSR,<br><br>median value	*p*-value
1	Kroon et al., 2008	31.0<br><br>(n=7, CV=0.38)	19.0<br><br>(n=7, CV=0.83)	0.249972
2	Nostro et al., 2011	5.1<br><br>(n=7, CV=0.95)	10.6<br><br>(n=7, CV=1.01)	**0.036443**
3	Loh et al., 2014	41.1<br><br>(n=5, CV=0.40)	15.1<br><br>(n=7, CV=0.93)	0.172526
4	Rezania et al., 2014/ Pagliuca et al., 2014; stage 3	62.6<br><br>(n=7, CV=0.13)	34.3<br><br>(n=3, CV=0.67)	0.094771
5	Rezania et al., 2014; stage 4	95.3<br><br>(n=6, CV=0.10)	43.65<br><br>(n=8, CV=0.51)	**0.001445**
6	Pagliuca et al., 2014; stage 4	92.4<br><br>(n=6, CV=0.04)	63.7<br><br>(n=5, CV=0.61)	**0.039630**

Supplementary Table 8: Differentiation to $PDX1^{+}$ progenitors of PSCs cultured in E8 compared to KSR conditions. Numbers of data points and coefficient of variation are indicated for data set. Significantly different comparisons are shown in bold.

© The Authors under the terms of the Creative Commons Attribution License http://creativecommons.org/licenses/by/3.0/, which permits unrestricted use, provided the original author and source are credited.

Protocol	Reference	H9<br><br>% PDX1 positive<br><br>(median value)	FiPS<br><br>% PDX1 positive<br><br>(median value)	*p*-value
1	Kroon et al., 2008	28.6 (n=3)	38.9 (n=4)	0.087698
2	Nostro et al., 2011	5.1 (n=3)	3.9 (n=4)	0.436144
3	Loh et al., 2014	28.0 (n=3)	44.1 (n=2)	0.123135
4	Rezania et al., 2014/ Pagliuca et al., 2014; stage 3	66.0 (n=3)	57.9 (n=4)	0.249718
5	Rezania et al., 2014; stage 4	95.9 (n=3)	94.6 (n=3)	0.442437
6	Pagliuca et al., 2014; stage 4	90.6 (n=3)	92.7 (n=3)	0.429211

Supplementary Table 9: Differentiation to $PDX1^+$ progenitors of H9 compared to FiPS cells. Numbers of data points are indicated for each median value.

© The Authors under the terms of the Creative Commons Attribution License http://creativecommons.org/licenses/by/3.0/, which permits unrestricted use, provided the original author and source are credited.

	Reference	% PDX1 positive (median)	*p*-value				
			Protocol 2	Protocol 3	Protocol 4	Protocol 5	Protocol 6
			Nostro et al., 2011	Loh et al., 2014	Rezania et al., 2014/Pagliuca et al., 2014; stage 3	Rezania et al., 2014; stage 4	Pagliuca et al., 2014; stage 4
1	Kroon et al., 2008	31.0 (n=7)	**0.000103**	**0.007744**	**0.000239**	**1.2x10E-06**	**1.6x10E-07**
2	Nostro et al., 2011	5.1 (n=7)		**0.000312**	**2.3x10E-09**	**2.2x10E-10**	**1.5x10E-12**
3	Loh et al., 2014	41.1 (n=5)			**0.000799**	**1.1E-05**	**1.5x10E-06**
4	Rezania et al., 2014/Pagliuca et al., 2014; stage 3	62.6 (n=7)				**4x10E-05**	**9.6x10E-07**
5	Rezania et al., 2014; stage 4	95.3 (n=6)					0.197981
6	Pagliuca et al., 2014; stage 4	92.6 (n=6)					

Supplementary Table 10: Differentiation to PDX1^{+} progenitors of PSC using different methods. P-values are shown for each pairwise comparison. Numbers of data points are indicated for each median value. Significantly different comparisons are shown in bold.

© The Authors under the terms of the Creative Commons Attribution License http://creativecommons.org/licenses/by/3.0/, which permits unrestricted use, provided the original author and source are credited.
